# Carbamoylated Nail Proteins as Assessed by Near-Infrared Analysis Are Associated with Load of Uremic Toxins and Mortality in Hemodialysis Patients

**DOI:** 10.3390/toxins12020083

**Published:** 2020-01-26

**Authors:** Sander De Bruyne, Jonas Himpe, Sigurd E. Delanghe, Griet Glorieux, Wim Van Biesen, Marc L. De Buyzere, Marijn M. Speeckaert, Joris R. Delanghe

**Affiliations:** 1 Department of Clinical Chemistry, Ghent University, 9000 Ghent, Belgium; sanderR.debruyne@ugent.be (S.D.B.); jonas.himpe@ugent.be (J.H.); 2 Department of Nephrology, Ghent University, 9000 Ghent, Belgium; sigurd.delanghe@ugent.be (S.E.D.); griet.glorieux@ugent.be (G.G.); wim.vanbiesen@ugent.be (W.V.B.); marijn.speeckaert@ugent.be (M.M.S.); 3 Department of Cardiology, Heart Center, Ghent University Hospital, 9000 Ghent, Belgium; marc.debuyzere@ugent.be; 4 Research Foundation Flanders, 1000 Brussels, Belgium

**Keywords:** carbamoylation, hemodialysis, keratins, mortality, nails, near-infrared spectroscopy, uremia, uremic toxins

## Abstract

Carbamoylation is an important risk factor for accelerated atherogenesis and mortality in patients undergoing hemodialysis (HD). We intended to explore whether carbamoylation as assessed by near-infrared (NIR) analysis of nail proteins is associated with (a) plasma concentrations of representative uremic toxins and (b) mortality in HD patients. A total of 53 healthy volunteers and 84 consecutive HD patients were enrolled in this cross-sectional cohort study. Standard laboratory methods were used to measure routine parameters, whereas levels of uremic toxins were determined using reversed-phase high-performance liquid chromatography (RP-HPLC). Spectra of distal fingernail clippings were obtained using an Avantes NIR spectrometer and processed using chemometric data analysis. The second derivative of the peak intensity at 1494 nm attributed to N-H amide bands from NH_2_ of carbamoyl (-CONH_2_) groups was higher in HD patients than in control subjects (*p* < 0.0001). Peak intensity levels were associated with age and plasma levels of representative uremic toxins. Cox-regression analysis revealed a significant association with all-cause mortality, even after adjustment for age. In conclusion, our data revealed that carbamoylation as assessed by NIR analysis of nail proteins is associated with plasma concentrations of uremic toxins and also with mortality in HD patients. Further research to explore whether it is a surrogate marker or a hard indicator of mortality risk is warranted.

## 1. Introduction

Chronic kidney disease (CKD) is a global public health problem with a varying prevalence ranging from 3–18%, depending on the region and population studied [1]. Patients with end-stage kidney disease (ESKD) suffer from an annual mortality of 15–20%, which is largely due to cardiovascular disease (CVD) [2]. Besides the traditional risk factors such as smoking, arterial hypertension and hypercholesterolemia [2], post-translational modifications (PTMs) of proteins are involved in the pathogenesis of CVD and play an important role in the progression of CKD. PTMs are covalent changes of proteins or peptides that are altered either by adding moieties to one or more amino acids or by proteolytic cleavage [3]. In theory, all proteins are susceptible to PTMs in vivo. However, the potential of each protein depends on several factors such as the accessibility and number of amino groups, as well as the protein half-life [4]. PTMs such as carbamoylation, glycation, oxidation, glycoxidation and methylation have different structural and functional effects [2].

The amounts of carbamoylated proteins are increased in hemodialysis (HD) patients when compared with normal subjects due to their chronic hyperuremia state [3,4]. An enhanced degree of carbamoylation has been identified as an important risk factor for accelerated atherogenesis and mortality in patients undergoing HD [2,5]. Several biomarkers have been proposed to assess the extent of protein carbamoylation in biological samples. Characteristic compounds such as homocitrulline often require specialized analytical techniques such as liquid chromatography–tandem mass spectrometry (LC-MS/MS) [6].

**Figure 1:**
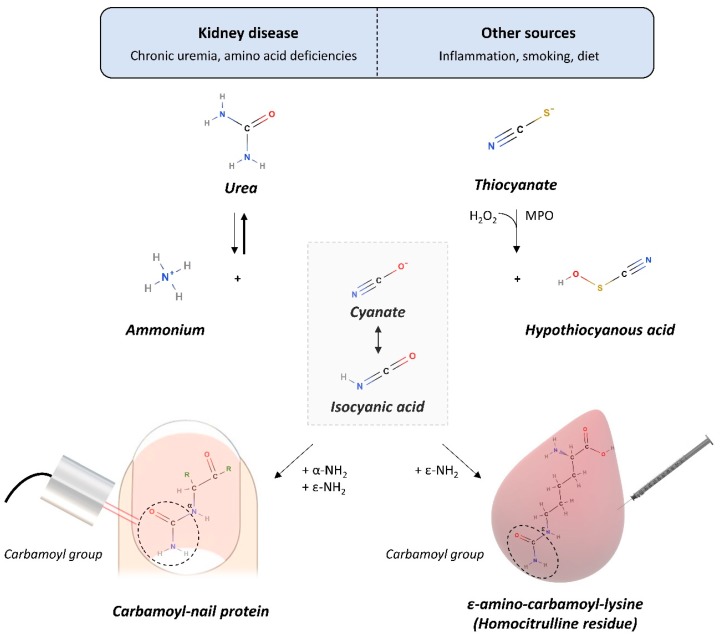
Carbamoylation is a nonenzymatic and irreversible post-translational modification during which a carbamoyl group (-CONH_2_) is added to proteins, peptides and amino acids by reaction with isocyanic acid, resulting in the formation of an irreversible covalent bond. Isocyanic acid originates mainly (1) from the spontaneous decompostion of urea into ammonium and cyanate, a reactive ion that is quickly converted to isocyanic acid, or (2) from myeloperoxidase (MPO)-catalyzed oxidation of thiocyanate at sites of inflammation, including atherosclerotic plaques, or (3) from environmental factors [2,10]. In chronic kidney disease, urea accumulates and elevates the concentration of isocyanic acid. The latter rapidly reacts with the α-amino group of peptides, proteins (e.g., nail keratins) or amino acids and with the ε-amino group of lysine, which results in the formation of homocitrulline (ε-amino-carbamoyl-lysine) [2].

The human nail plate can be seen as a specialized keratinous skin appendage, which is in close contact with the capillary bed of the fingers and toes. While a slower diffusion rate of molecules is found in the nail matrix compared to serum, nail growth is a slow process (complete replacement takes 6–9 months on average) that allows the occurrence of chemical reactions between nail keratins and small molecular mass compounds such as glucose and urea [7,8]. Nail keratins have the potential to accumulate PTMs such as carbamoylation due to their high protein content (80% of total mass) and long half-life (Figure 1) [2], which may eventually lead to changes in the near-infrared (NIR) spectrum of the nail [7].

NIR spectroscopy is a simple, quick, cost-effective and noninvasive technique that uses the 780–2526 nm wavelength range of the electromagnetic spectrum. This region is mainly composed of absorption bands related to overtones and combinations of fundamental vibrations of -CH, -NH, -OH and -SH functional groups. NIR spectroscopy has major advantages over other analytical techniques. It is able to provide information on the chemical composition of biological samples from one single spectrum in a noninvasive and ultrafast way, without the need for sample preparation [9]. Therefore, we intended to explore whether carbamoylation as assessed by NIR analysis of nail proteins is associated with (a) plasma concentrations of uremic toxins and (b) mortality in HD patients.

## 2. Results

### 2.1. The Peak Intensity at 1494 nm Is Significantly Higher in Hemodialysis Patients Versus Controls

A comparison of demographic characteristics, clinical characteristics and laboratory measurements in controls and HD patients can be found in Table 1. 

**Figure 2:**
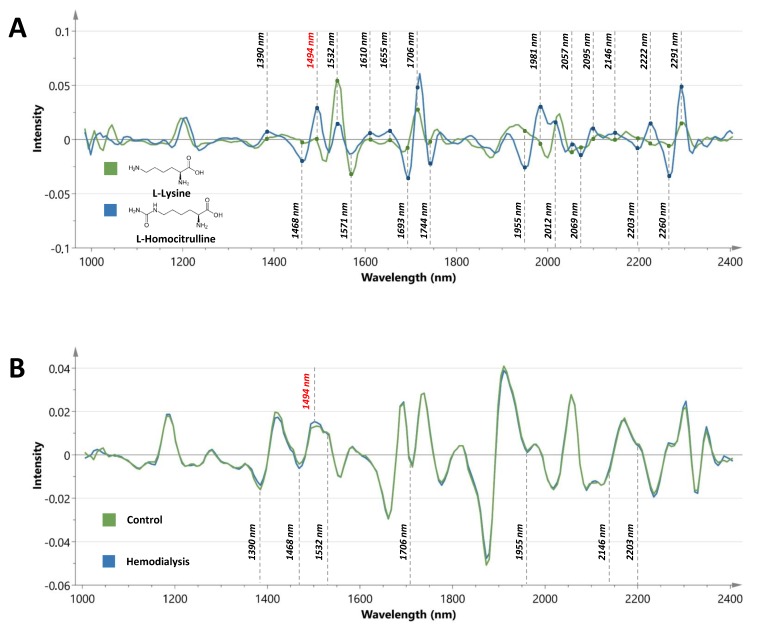
Spectral signature of carbamoylation. (**A**) Second derivative spectra of L-lysine (green line) and L-homocitrulline (blue line) powder. Characteristic spectral peaks are indicated. (**B**) Second derivative of the median spectra from controls (*n* = 53, green line) and hemodialysis patients (*n* = 84, blue line). Concordant spectral changes with the differences observed in the spectra of lysine and homocitrulline are highlighted (1494 nm region in red).

After using standard normal variate (SNV), Savitsky–Golay smoothing (SG, 7 points) and second derivative conversion as preprocessing steps on the spectral range from 1038 to 2355 nm, preliminary principal component analysis (PCA) results showed clear discrimination and clustering of healthy subjects and HD patients. The same preprocessing steps were applied to the spectra of lysine and homocitrulline powder (Figure 2A). Table 2 provides an overview of the characteristic spectral peaks observed in the spectrum of homocitrulline together with associated functional groups, differences with the spectrum of lysine, concordance with spectral changes observed in controls and HD patients and the potential influence of diabetes mellitus (DM). From the 21 selected regions, 10 peaks showed no significant differences between controls and HD patients. Eight regions showed spectral changes in line with the spectra of homocitrulline and lysine and the differences observed between healthy subjects and HD patients; three regions showed discordant results. 

**Figure 3:**
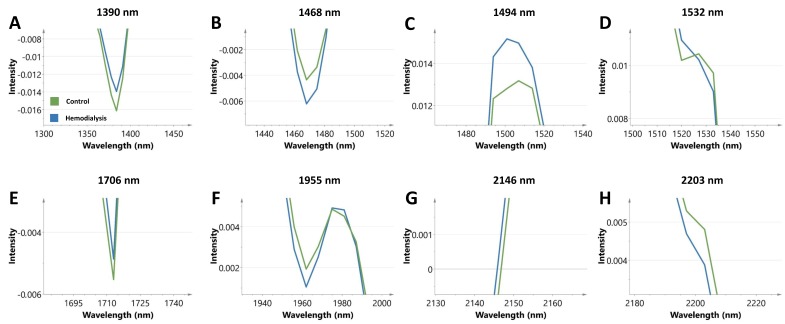
Close-up on the median second derivative spectra from controls (*n* = 53, green line) and hemodialysis patients (*n* = 84, blue line) of the eight regions, characterized by changes in line with the spectra of homocitrulline and lysine. (**A**) 1390 nm; (**B**) 1468 nm; (**C**) 1494 nm; (**D**) 1532 nm; (**E**) 1706 nm; (**F**) 1955 nm; (**G**) 2146 nm; and (**H**) 2203 nm.

Figure 2B represents the median spectra of controls and HD patients with marking of concordant regions, and Figure 3 provides a close-up on the latter. None of the eight regions were hampered by a confounding effect of DM. Receiver operating characteristic (ROC) curve analysis was used to further explore the diagnostic efficiency of the eight concordant spectral markers and yielded the best area under the curves (AUCs) for the peak intensities at 1468 nm (AUC: 0.90, *p* < 0.0001), a region associated with the N-H combination band from RCONH_2_ groups, and at 1494 nm (AUC: 0.82, *p* < 0.0001), a region linked to N-H amide bands from NH_2_ groups. Comparison of age-matched controls did not reveal significant differences between control samples stored for 438–897 days (*n* = 12, median value intensity: 0.013, IQR: 0.011–0.01) and fresh obtained samples (*n* = 12, median value intensity 0.012, IQR 0.011–0.012) for the peak intensity at 1494 nm (*p* = 0.13). However, in case of the peak intensity at 1468 nm, significantly higher intensity values were found for the fresh obtained samples (median value intensity: −0.0035, IQR: −0.0038 to −0.0021) compared to the stored samples (median value intensity: −0.0047, IQR: −0.0055 to −0.0034). Based on the latter, the spectral marker at 1494 nm was selected as the most representative marker in the assessment of carbamoylated fingernail proteins and used for further analysis. The procedure showed a good within-run precision (coefficient of variation (CV) = 4.4%) for the peak intensity at 1494 nm.

### 2.2. The Spectral Marker at 1494 nm Is Associated with Age and Uremic Toxins

Multiple linear regression analysis was run to reveal associations between the peak intensity at 1494 nm (milliunits) and age, presence of DM and uremic toxins. In separate models, age and several uremic toxins (total indoxyl sulfate (IS), total p-cresylglucuronide (PCG), free PCG, free hippuric acid (HA) and total HA) added significantly to the prediction. The presence of DM did not appear to be a confounding factor. Table 3 provides an overview of the different multiple regression analysis models with the peak intensity at 1494 nm as a dependent variable.

### 2.3. The Spectral Marker Has Prognostic Utility among Hemodialysis Patients

**Figure 4:**
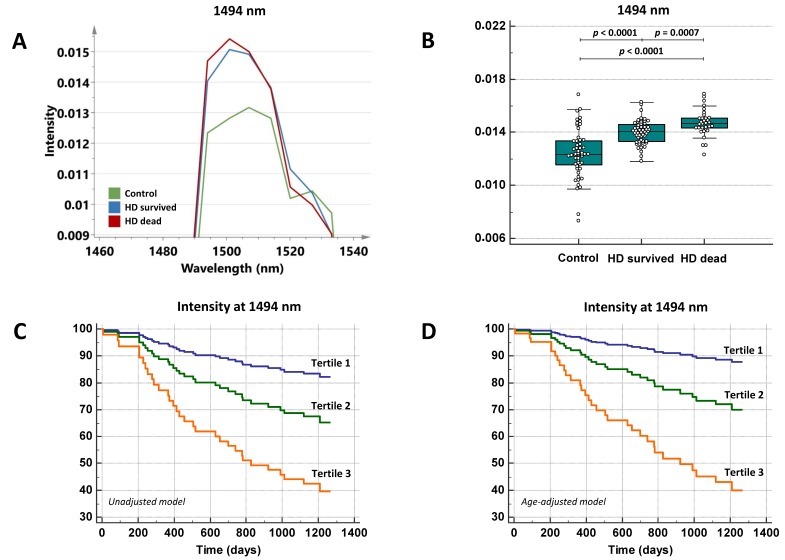
(**A**) Close-up on the second derivative spectra of the 1494 nm region in control subjects (*n* = 53, green line), hemodialysis (HD) patients who survived (*n* = 53, blue line) and HD patients who died during the 3-year follow-up period (*n* = 31, red line). (**B**) Box-and-whisker plots illustrating differences in peak intensities between controls, HD patients who survived and HD patients who died during the follow-up period. The *p*-values represent results obtained from Mann–Whitney U tests. Unadjusted (**C**) and age-adjusted (**D**) cox-regression survival curves for increasing tertiles of the spectral marker at 1494 nm in the group of hemodialysis patients (*n* = 84).

Univariate Cox regression analysis revealed that age (hazard ratio (HR) = 1.065, 95% confidence interval (CI) = 1.028–1.10, *p* = 0.0005), history of CVD (HR = 2.81, 95% CI = 1.22–6.50, *p* = 0.016), free PCG levels (HR = 11.67, 95% CI = 3.67–37.12, *p* < 0.0001), total PCG levels (HR = 8.97, 95% CI = 3.13–25.69, *p* < 0.0001), free IS levels (HR = 133.00, 95% CI = 3.17–5584.22, *p* = 0.011), total HA levels (HR = 1.12, 95% CI = 1.01–1.24, *p* = 0.041) and free HA levels (HR = 1.20, 95% CI = 1.019–1.41, *p* = 0.030) were significantly associated with the risk of death. The peak intensity at 1494 nm appeared to be significantly higher among HD patients who died during the follow-up period (*n* = 31) than among HD patients who survived (*n* = 53, *p* = 0.0007, Figure 4A,B). 

Table 4 shows the association of the spectral marker at 1494 nm with the risk of death in an unadjusted model and an age-adjusted model. A significant association between the intensity at 1494 nm and all-cause mortality was found even after adjustment for age. Figure 4 shows the survival plots of increasing tertiles of the spectral marker for the unadjusted model (Figure 4C) and the model adjusted for age (Figure 4D).

## 3. Discussion

In the present paper, we identified for the first time several spectral features related to carbamoylation in distal fingernail clippings of HD patients. The intensity at 1494 nm, attributed to N-H amide bands from NH_2_ groups, was significantly associated with age and plasma levels of representative uremic toxins. More important, this intensity was also significantly associated with mortality during the 3-year follow-up period, even after adjusting for age. Our study demonstrates that measuring the load of carbamoylated nail keratins through NIR analysis is a promising tool to identify HD patients at risk due to uremic load. Further research to corroborate NIR as a hard indicator would yield an easy-to-use indicator of uremic load that can be used to steer dialysis treatment. 

Our data point out an association between concentrations of representative uremic toxins and intensity of NIR analysis. Further research is needed to explore whether more intensive dialysis regimens resulting in a decrease of uremic load would also result in changes in carbamoylation as assessed by NIR. If this would be the case, NIR would be a good tool to assess the impact of changes in dialysis regimen. The current method Kt/V_urea_ is recently progressively criticized as it is not representative of uremic load and is also not associated with mortality [11,12]. Since our data revealed that the spectral marker appeared to be an independent risk factor for mortality and is characterized by significant associations with uremic toxins, we can hypothesize that lowering the level of uremic toxins could lead to a decrease in its peak intensity and subsequently a lower mortality risk. Promising for our hypothesis is that preliminary results obtained from HD patients before and after kidney transplantation revealed a clear decrease of the spectral marker (data not shown). In contrast, in a cohort of 21 patients on a stable HD regimen, and thus a stable uremic load, repetitive sampling demonstrated no changes of spectral intensity over time (data not shown). 

Direct NIR analysis of fingernails presents attractive features such as being ultrafast (<10 s analysis time) and reagent-free. Moreover, handheld miniaturized NIR spectrometers offer great potential for on-site monitoring [13,14]. Therefore, this approach is much easier than analytical techniques classically used in the assessment of carbamoylated proteins such as LC-MS/MS [6]. Furthermore, in contrast to serum or plasma, the nail matrix does not require important preanalytical precautions, as even prolonged storage (4 °C) did not reveal significant differences for the peak intensity at 1494 nm. In addition, nail keratins are hardly affected by intraindividual variability compared to other plasma protein fractions. However, the methodology has to be standardized in the future to introduce this analytical technique in clinical diagnostics. In addition, nail abnormalities such as trophic disturbances and onychomycosis might be associated with spectral properties that interfere in our regions of interest. Since these abnormalities are much more common at the toenail level, fingernails are the preferred matrix to avoid or minimize potential spectral interferences [14,15]. 

We did not perform LC-MS/MS, mostly considered the gold standard, to quantify the degree of carbamoylation. However, while the LC-MS/MS technique has already been used for the quantification of carbamoylated albumin and homocitrulline in serum or tissues [16,17], in fact no methods that have been validated as gold standard are currently available for quantification of carbamoylated nail keratins. Comparison of our NIR results with carbamoylation-derived products in serum is not expected to provide essential information since fingernails are characterized by a much longer protein half-life compared to cross-sectional samplings of blood markers. Nevertheless, our confidence that the NIR spectra reveal carbamoylation is supported by the fact that the most discriminative regions fall in biochemically very acceptable regions and that these regions are largely in concordance with characteristic differences observed in the spectra of homocitrulline and lysine. Furthermore, the NIR spectral intensity at 1494 nm is highly associated with age, whereas protein carbamoylation is a hallmark of ageing [18,19]. Seemingly paradoxical, no significant association was found between the spectral marker and levels of urea after adjustment for age. However, regarding the major differences in half-life time between carbamoylated nail keratins and urea, a single snapshot measurement cannot be perceived as being fully representative for the average urea levels observed during the duration of a complete nail replacement (6–9 months). Furthermore, it has been widely accepted that serum urea levels are poorly reflecting concentrations of other, more toxic, uremic retention products [12].

No significant confounding effect of the presence of DM (i.e., glycation) could be identified. The latter is an important finding for several reasons. First of all, CKD and DM are diseases that accelerate protein molecular ageing through carbamoylation and glycation reactions, respectively, on the same protein amino groups, and, consequently, in vivo competition may arise. However, previous research showed that carbamoylation exerts a more potent competitive effect on glycation than does glycation on carbamylation [20]. Furthermore, due to the loss of renal function and inability of the kidney to excrete advanced glycation end-product (AGE) precursors in HD patients, concentrations of dicarbonyl metabolites are increased, which leads to an accumulation of AGEs [3]. However, it has to be mentioned that next to carbamoylation and glycation, many other PTMs (e.g., oxidation) occur during the biological life of proteins [21,22]. The latter is a plausible explanation for the fact that not all spectral regions showed changes in line with the differences observed in the spectra of homocitrulline and lysine. 

## 4. Conclusions

In conclusion, load of carbamoylated nail proteins as assessed by NIR spectroscopy is associated with uremic toxins and is an important risk indicator for mortality in HD patients. Further research to explore whether it is a surrogate marker or a hard indicator of mortality risk is warranted. If this could be demonstrated, the simple and highly economical character of the proposed method makes it a well suited and innovative additional tool in the future management of HD patients.

## 5. Materials and Methods 

### 5.1. Research Participants and Specimen Collection

For the cross-sectional cohort, a total of 53 healthy volunteers (male/female ratio: 18/35; median age: 34.8 years) and 84 consecutive HD patients (male/female ratio: 52/32; median age: 73.7 years) were enrolled in this study from December 2015 till September 2018. HD patients were recruited at the Department of Nephrology of the Ghent University Hospital, Ghent, Belgium and were treated with a regular 4-h conventional hemodialysis session (blood flow rate: 300–350 mL/min and dialysate flow rate with an autoflow factor of 1.5) three times weekly. Distal fingernail specimens were obtained with standard nail clippers and stored at 4 °C. The presence of nail polish was an exclusion criteria. Blood samples (serum and plasma) were collected, centrifuged, aliquoted and stored at −80 °C until analysis. The study was approved by the local Ethics Committee (Ethical approval recruitment of control subjects: 2010/033; B67020107926, Ethical approval recruitment of dialysis patients: 2015/0932; B670201525559). All participants signed a written informed consent.

### 5.2. Study Parameters

In ESKD patients, blood was taken before the start of a midweek HD session. In the overall group, routine laboratory parameters were determined in serum using the Alinity c system (Abbott, Chicago, IL, US). C-reactive protein was analyzed using a particle enhanced immunoturbidimetric assay. Total protein, creatinine and urea were quantified using kinetic colorimetric assays based on the biuret, Jaffé (compensated rate-blanked picrate assay) and Talke and Schubert’s methods, respectively. As described earlier [23,24], seven uremic toxins were determined by reversed-phase high performance liquid chromatography (RP-HPLC): the protein bound solutes indoxyl sulfate (IS, 213 Da), p-cresylsulfate (PCS, 187 Da), p-cresylglucuronide (PCG, 284 Da), indole acetic acid (IAA, 175 Da), 3-carboxy-4-methyl-5-propyl-2-furanpropionic acid (CMPF, 240 Da), hippuric acid (HA, 179 Da) and the non-protein-bound uric acid (UA, 168 Da). Prior to analysis, samples were deproteinized by heat denaturation (95 °C, 30 min) and afterwards placed on ice for 10 min. Afterwards, plasma samples were centrifuged (7379 × *g*, 10 min) and filtered (3615 × *g*, 20 min, 21 °C) using a 30 kDa cut-off molecular filter (Amicon Ultra 0.5 mL Filters, Merck KGaA, Darmstadt, Germany). To obtain free fractions, the denaturation process was preceded by filtration (Centrifree filter device, Millipore Billerica, MA, USA). While PCG and PCS (λ_exc_ = 265 nm, λ_em_ = 290 nm) and IAA and IS (λ_exc_ = 280 nm, λ_em_ = 340 nm) were analyzed by fluorescence detection, HA and CMPF were determined by UV detection at 254 nm, and UA at 300 nm. In the group of HD patients some additional markers were determined. White blood cells, red blood cells, thrombocytes, hemoglobin and hematocrit were determined on a XE-5000 system (Sysmex, Bornbarch, Germany). Erythrocyte sedimentation rate was analyzed on a Starrsed RS system (Sysmex, Bornbarch, Germany). While liver enzymes (aspartate aminotransferase and alanine transaminase) were determined using a kinetic colorimetric assay on the Alinity c system, ferritin concentrations were detected using a chemiluminescent microparticle immunoassay on an Alinity i system (Abbott). In addition, comorbidity was assessed using the Stoke study scoring system (Davies score) [25,26]. 

### 5.3. Near-Infrared Spectroscopy

Spectral data were obtained at ambient temperature using a NIR spectrometer (AvaSpecNIR256-2.5-HSC, Avantes, Apeldoorn, the Netherlands) equipped with extended InGaAs array technology. Fingernails often have a convex shape, which has consequences for measurements based on reflectance spectroscopy. When a radiation beam hits a surface, it can be either transmitted, absorbed or reflected. The relative amount of transmission and reflection depends largely on the refractive index of the sample, air and angle of incidence. To obtain a sufficient amount of reflected beams onto the detector and consequently sufficient signal intensity, the position of the radiation beam onto the convex nail sample has to be carefully selected. In order to bypass this potential pitfall and exclude spectral variations caused by the angle of incidence, an immobilized 50 mm integrating sphere was used (AvaSphere-50-LS-HAL-6-S1, Avantes). An integrating sphere works as a uniform light collector independent of sample orientation and allows analysis of extended sample areas [27]. Before nail analysis, visible dirt was removed using a standard pincet. Ventral surfaces of the distal fingernail clippings were oriented towards the integrating sphere and spectral data were recorded across the spectral range 1038–2354 nm at a spectral resolution of 13 nm (128 co-added scans). All samples were analyzed in batch to minimize external variabilities. In addition, NIR spectra of L-lysine powder (≥98%, Sigma-Aldrich, St. Louis, Missouri, USA) and its carbamoylated analogue, i.e., L-homocitrulline (≥95%, Santa Cruz Biotechnology, Dallas, TX, USA) were recorded. An in-depth comparison of the spectra was performed to identify spectral changes related to the addition of a carbamoyl moiety (-CONH_2_) on protein or amino acid functional groups. Since previous research of our group showed that glycated nail keratins are able to induce spectral changes in the NIR region [27], the potential influence of glycation in the zones of interest was investigated by comparison of the peak intensities between HD patients with DM (*n* = 30) and HD patients without DM (*n* = 54). To investigate a potential influence of storage time on spectral intensities, age-matched control samples stored for 438–897 days at 4 °C (*n* = 12, median age 42.0, interquartile range (IQR) 25.0–42.5) were compared with fresh obtained samples (*n* = 12, median age: 39.7 years, IQR: 25.5–44.2 years).

### 5.4. Spectral Data Analysis

Spectral data analysis was carried out using SIMCA 15 (Umetrics, Umeå, Sweden). To standardize spectroscopic signals and remove irrelevant scatter light, several preprocessing steps were employed [14]. NIR spectra were mean-centered, normalized using SNV method and converted to their derivatives with a SG algorithm. We used SNV to remove additive baseline offset variations and multiplicative scaling effects due to differences in sample density and sample-to-sample measurement variations, and SG smoothing to minimize spectral noise. We used derivatives to enhance spectral discrimination by highlighting small-scale differences between similar spectra, reducing baseline effects and causing spectral resolution enhancement by resolving overlapping spectral bands [27,28]. The rate of change of intensity with respect to wavelength (λ) was examined by the first derivative, whereas the second derivative was used to assess alterations in the rate of change of intensity, an effective method for removing sloping baselines [14,27]. After pretreatment of spectra, PCA was performed to sort spectra and to get an impression of the complexity, heterogeneity and similarity of the dataset and thereby unravelling spectral differences between healthy subjects and HD patients [13,29].

### 5.5. Statistical Analysis

Statistical data analysis was performed using MedCalc version 18.11 (MedCalc Software, Mariakerke, Belgium). Normality of distributions was tested by the Kolmogorov–Smirnov test. Values are expressed as median with IQR. Differences between two groups were assessed using the Mann–Whitney U test. Multiple linear regression analysis was performed to unravel the association of the peak intensity at 1494 nm with age, uremic toxins and the presence of DM (confouding factor). To avoid collinearity bias, highly intercorrelated variables were not included in the same model. Unadjusted and age-adjusted analyses of mortality were generated using Cox proportional-hazards regression on the relative intensities as a continuous variable. A *p* value < 0.05 was considered a priori to be statistically significant. To test the reproducibility of our method, fingernail clippings of three randomly selected healthy subjects and three HD patients were analyzed 10 times to calculate the within-run CV. ROC curve analysis was performed to unravel the discriminative potential of individual spectral markers.

## Figures and Tables

**Table 1:** Demographic, clinical and laboratory measurements in healthy subjects and HD patients.

Variable	Control (*n* = 53)	HD (*n* = 84)
**Demographic and Clinical**		
Age (yr)	34.8 (27.5–43.5)	73.7 (62.0–81.3)
Male/female ratio	18/35	52/32
Caucasian (%)	100	100
History of CVD (%)	0	59.5
Diabetes mellitus (%)	0	35.7
BMI (kg/m^2^)	23.2 (20.6–25.7)	26.1 (23.4–29.1)
**Laboratory**		
Creatinine (mg/dL)	0.9 (0.8–1.0)	7.0 (5.0–8.8)
CRP (mg/L)	1.1 (0.7–2.8)	4.7 (1.9–10.8)
Total protein (g/L)	74.0 (69.9–81.5)	66.0 (62.5–69.6)
Urea (mg/dL)	26.7 (22.8–31.7)	99.0 (74.5–127.0)
IS total (mg/dL)	0.06 (0.04–0.08)	1.8 (1.0–2.6)
IS free (mg/dL)	0.001 (0.0005–0.001)	0.09 (0.05–0.2)
PCS total (mg/dL)	0.2 (0.1–0.3)	3.2 (2.0–4.2)
PCS free (mg/dL)	0.008 (0.003–0.01)	0.2 (0.1–0.3)
PCG total (mg/dL)	0.001 (0.001–0.001)	0.2 (0.1–0.4)
PCG free (mg/dL)	0.001 (0.001–0.001)	0.2 (0.08–0.4)
IAA total (mg/dL)	0.03 (0.02–0.03)	0.1 (0.1–0.2)
IAA free (mg/dL)	0.001 (0.001–0.002)	0.04 (0.03–0.06)
CMPF (mg/dL)	0.05 (0.01–0.1)	0.5 (0.3–0.9)
HA total (mg/dL)	0.07 (0.05–0.1)	2.7 (1.0–4.7)
HA free (mg/dL)	0.02 (0.006–0.04)	1.1 (0.5–2.6)
UA (mg/dL)	4.1 (3.4–5.3)	5.4 (4.8–6.4)

Abbreviations: BMI, body mass index; CMPF, 3-carboxy-4-methyl-5-propyl-2-furanpropionic acid; CRP, C-reactive protein; CVD, cardiovascular disease; HD, hemodialysis; HA, hippuric acid; IAA, indole acetic acid; IS, indoxyl sulfate; PCG, p-cresylglucuronide; PCS, p-cresylsulfate; UA, uric acid; Yr, year. Data are presented as median (interquartile range).

**Table 2:** Overview of the characteristic spectral peaks observed in the spectrum of homocitrulline with associated functional groups, differences with the spectrum of lysine, concordance with spectral changes observed in controls (*n* = 53) and hemodialysis (HD, *n* = 84) patients and influence of diabetes mellitus (DM).

Wavelength (nm)	Associated Functional Groups	Homocit vs. lys	Control (Intensity)	HD (Intensity)	*p*	In Line	Sign. Influence DM (*p*)
1390	C-H bands	increase	−0.012	−0.011	<0.0001	yes	No (0.24)
1468	CONH_2_ (N-H combination band)	decrease	−0.0044	−0.0062	<0.0001	yes	No (0.42)
1494	N-H amide	increase	0.012	0.014	<0.0001	yes	No (0.90)
1532	N-H amide	decrease	0.0097	0.0090	0.0001	yes	Nearly sign. decrease (0.069)
1571	N-H amide	increase	0.00034	−0.000043	0.0001	no	No (0.72)
1610	C=O/N-H combination	increase	−0.00032	−0.0011	<0.0001	no	No (0.91)
1655	C-H methyl	increase	−0.024	−0.026	0.0006	no	Nearly sign. increase (0.073)
1693	CONH_2_ (N-H and C=O)	decrease	-	-	n.s.	-	-
1706	C-H methyl	increase	−0.0016	−0.00054	0.0003	yes	No (0.80)
1744	CONH_2_ (C=O hydrogen bonded to N-H)	decrease	-	-	n.s.	-	-
1955	CONH_2_ (N-H combination band)	decrease	0.0040	0.0029	<0.0001	yes	No (0.83)
1981	CONH_2_ (N-H amide II)	increase	-	-	n.s.	-	-
2012	CONH_2_ (N-H/C-N combination band)	-	-	-	n.s.	-	-
2057	CONH_2_ (N-H/C=O amide)	increase	-	-	n.s.	-	-
2069	N-H amide (N-H deformation)	decrease	-	-	n.s.	-	-
2095	N-H	increase	-	-	n.s.	-	-
2146	N-H/C-N/C=O	increase	−0.00076	0.00022	<0.0001	yes	No (0.60)
2203	CONH_2_ (N-H and C=O)	decrease	0.0048	0.0039	=0.010	yes	No (0.39)
2222	N-H combination	increase	-	-	n.s.	-	-
2260	CONH_2_ (N-H and C=O)	decrease	-	-	n.s.	-	-
2291	CONH_2_ (C=O hydrogen bonded to N-H)	increase	-	-	n.s.	-	-

Abbreviations: homocit, homocitrulline; lys, lysine; nm, nanometer; n.s., nonsignificant; sign., significant. The *p*-values represent the results of Mann–Whitney U tests.

**Table 3:** Multiple regression models with the peak intensity at 1494 nm (milliunits) as a dependent variable.

Model	r^2^ (p)	Retained Variables	β (SE)	*p*
Age, DM and urea	0.24 (<0.001)	Age (yr)	0.038 (0.0060)	<0.0001
Age, DM and IS total	0.27 (<0.001)	Age (yr)	0.028 (0.0067)	0.0001
		IS total (mg/dL)	0.34 (0.12)	0.0058
Age, DM and PCG total	0.26 (<0.001)	Age (yr)	0.029 (0.0072)	0.0001
		PCG total (mg/dL)	1.29 (0.58)	0.028
Age, DM and PCG free	0.26 (<0.001)	Age (yr)	0.029 (0.0071)	0.0001
		PCG free (mg/dL)	1.41 (0.63)	0.027
Age, DM and HA total	0.27 (<0.001)	Age (yr)	0.029 (0.0067)	<0.0001
		HA total (mg/dL)	0.14 (0.050)	0.0063
Age, DM and HA free	0.26 (<0.001)	Age (yr)	0.030 (0.21)	<0.0001
		HA free (mg/dL)	0.21 (0.082)	0.013

Abbrevations: DM, diabetes mellitus; HA, hippuric acid; IS, indoxyl sulfate; PCG, p-cresylglucuronide; SE, standard error; Yr, year.

**Table 4:** Unadjusted and age-adjusted Cox regression analysis for all-cause mortality with the intensity at 1494 nm (milliunits) entered as a continuous variable in all hemodialysis patients (*n* = 84).

Models of Patient Survival (Events *n* = 31)	HR	95% CI	*p*
**Unadjusted Model**			
Intensity at 1494 nm (milliunits)	1.81	1.26–2.58	0.0012
**Age-Adjusted Model**			
Intensity at 1494 nm (milliunits)	2.05	1.22–3.01	0.0001
Age (yr)	1.07	1.03–1.11	0.0002

Abbreviations: CI, confidence interval; HR, hazard ratio; yr, year.
